# Blockchain-based zero trust networks with federated transfer learning for IoT security in industry 5.0

**DOI:** 10.1371/journal.pone.0323241

**Published:** 2025-06-06

**Authors:** Ankita Sharma, Shalli Rani, Wadii Boulila

**Affiliations:** 1 Chitkara University Institute of Engineering and Technology, Chitkara University, Rajpura, Punjab, India; 2 Robotics and Internet-of-Things Laboratory, Prince Sultan University, Riyadh, Saudi Arabia; Cardiff Metropolitan University - Llandaff Campus: Cardiff Metropolitan University, UNITED KINGDOM OF GREAT BRITAIN AND NORTHERN IRELAND

## Abstract

The rise of Industry 5.0 focuses on merging advanced intelligence, automation, and human-centered teamwork in industrial settings. However, keeping interconnected IoT networks secure is still a challenging problem. This paper proposes a new security framework that combines Blockchain, Federated Transfer Learning, and zero trust network (ZTN) principles to improve IoT security in Industry 5.0. Blockchain is a decentralized ledger that ensures secure data sharing and protects model updates. Federated Transfer Learning allows model training across distributed IoT devices to keep data private. The ZTN approach enforces strict access rules, assuming that no entity is trusted by default. The proposed framework offers a scalable and resilient solution to protect next-generation industrial IoT networks, using Blockchain for data security, transfer learning for adaptability, and ZTN for strict access control. The ZTN architecture strengthens security by checking every access request and keeping the IoT system safe. The experimental results show good performance of the proposed method, with better accuracy, precision, recall, and F1 scores. The model achieved an accuracy of 0.85, 0.88, and 0.87 for learning rates of 0.01, 0.001, and 0.0001, respectively, at 100 epochs. The precision values reached 0.84, 0.87, and 0.86, while the recall scores were 0.82, 0.86, and 0.85, respectively. The F1-scores were recorded at 0.83, 0.86, and 0.85, which confirms the robustness of our model.

## 1 Introduction

It is anticipated that Next-Generation Networks (NGNs) will be able to efficiently manage an extensive array of devices, industries, technologies, and services. The presence of heterogeneity significantly increases the complexity of managing network infrastructure [1]. Software-defined networking (SDN) and network function virtualization (NFV) have gained traction as methods for managing the fluctuating demand for network resources. Novel approaches are needed to address challenges in ensuring end-to-end Quality of Experience (QoE) and efficient automated resource management in network softwarization [2]. These factors are very important because virtualized resources are linked to physical entities that may be connected to different service providers. The 5th industrial revolution offers organizations new chances to enhance customer value by leveraging communication and computing technologies that are enabled [3]. Cloud-native gateways provide businesses with new possibilities to utilize this technology for expansion and financial success through potential business models. It is essential for all key stakeholders, including prominent telecoms and vertical companies, to collaborate to achieve the necessary economic impact and ensure fair recompense for everyone engaged. The ZTN framework is a high-tech system that aims to do all operational tasks, such as provisioning, monitoring, optimization, delivery, deployment, planning, design, and more without much help from people [4]. By employing ZTNs, network management solutions can offer inherent security. The widespread use of IoT devices and ZTNs’ reliance on edge and cloud-based services are the primary sources of current security risks. IoT devices are vulnerable to hacking [5] because they lack security measures during their physical installation in ZTNs. Furthermore, using an unprotected conduit for communication creates the possibility for many security and privacy breaches to occur inside the network [6]. Hence, it is crucial to examine the most effective strategies to address the previously listed risks and attacks on ZTNs [7]. DL can uncover insights from large amounts of heterogeneous and unorganized ZTN data. Another benefit is that it allows for the automated identification of hidden patterns from large amounts of data, eliminating the requirement for human involvement [8]. To prevent undiscovered fraudulent transactions, intrusion detection systems (IDS) can be strategically installed or redundantly constructed within a network. The primary objective of an IDS is to analyze and categorize network traffic [9]. It is highly efficient at differentiating malicious from authorized network activity, thereby assisting in removing undesired traffic [10]. Unlike machine learning-based IDS, deep learning-based IDS can improve overall accuracy and support a broader range of applications [11]. Research on establishing blockchain and DL-based solutions for secure data exchange, specifically for ZTNs, is still early [12]. In addition, there is a scarcity of research studies investigating the hazards linked to transitioning from traditional to ZTN networks [13]. The studies cited in [14] and [15] provide a variety of frameworks or approaches that can be used to develop IDS in ZTNs using machine learning or deep learning. However, detecting intrusions using standalone machine learning or deep learning-based IDS is difficult for most ZTN applications. The time series data in these applications is usually very long, and there are a lot of features. Because the features are not linear or stationary, they are organized into many layers. Traditional AI/ML-based security models in IoT networks face several challenges, particularly in the context of ZTN. These models often rely on static training datasets and predefined feature extraction, making them less effective in detecting sophisticated cyber threats that evolve over time. Furthermore, IoT networks generate vast amounts of heterogeneous, time-series data, posing scalability and adaptability challenges for conventional AI/ML models. Manual feature engineering, a crucial step in traditional models, is prone to human error, further compromising detection accuracy. Deep learning (DL)-based approaches have emerged as a promising solution, offering automated feature extraction and real-time adaptation to new threats. But using DL models in IoT-based ZTN settings needs a strong security system to make sure that data and model updates are safe [16]. Our proposed framework integrates blockchain, federated transfer learning, and ZTN principles to address these challenges. We ensure secure model updates and data integrity by leveraging blockchain’s decentralized and tamper-proof properties. Federated Transfer Learning enables efficient adaptation of security models across diverse IoT devices without exposing sensitive data. The ZTN framework further reinforces security by enforcing strict access control policies, ensuring that no entity is inherently trusted. This multidisciplinary approach significantly enhances the resilience, scalability, and adaptability of IoT security in Industry 5.0.

### 1.1 Research gaps

Despite significant advances in IoT security, several challenges remain unaddressed. Existing centralized security frameworks create scalability and reliability issues, as they are prone to single points of failure and large-scale cyberattacks. While machine learning-based IDS have improved threat detection, they still require frequent manual retraining and lack adaptability to emerging cyber threats [17]. FL has been proposed as a solution to decentralized training, but existing FL-based security approaches do not incorporate mechanisms to ensure the integrity of model updates. Blockchain technology offers tamper-proof data storage and secure model exchange; however, its potential in federated learning environments remains underexplored. Additionally, ZTN provides a robust security model by enforcing strict access control policies, yet its integration with federated learning for IoT security is still in its infancy. To fill in these gaps, our research suggests a Blockchain-Enhanced Federated Transfer Learning framework that uses ZTN ideas to create a secure, decentralized, and flexible system for IoT settings in Industry 5.0.

### 1.2 Our contributions

- The experimental setup and results discussed in this paper highlight the significant improvements in security and efficiency achieved through this integrated approach.
- By adopting a federated learning model, the system allows for the collective enhancement of security measures while preserving the privacy and operational autonomy of individual devices.
- We address the challenges of scalability and computational overhead, providing a roadmap for future enhancements and the potential for broader application across various IoT domains.
- The results demonstrate the progression of the model accuracy over training cycles. The study also highlights the influence of different learning rates and epochs on a variety of model metrics.
- The model achieved an accuracy of 0.85, 0.88, and 0.87 for learning rates of 0.01, 0.001, and 0.0001, respectively, at 100 epochs. Precision values reached 0.84, 0.87, and 0.86, while recall scores were 0.82, 0.86, and 0.85, respectively. The F1-scores were recorded at 0.83, 0.86, and 0.85, confirming the robustness of our model.

Section 2 discusses the attacks on ZTN; the next section discusses the overview and security architecture of ZTN; Section 4 discusses the proposed methodology with mathematical formulations, followed by the next section of results and discussions. The last section discusses the conclusion.

## 2 Attacks on ZTN

The following section discusses the respective layers of ZTN along with possible attacks on the layers.

### 2.1 Perception layer

The perception layer is also referred to as the hardware layer. The system comprises a wide array of actuators and sensors with constrained resources [18]. The many components establish communication among themselves through multiple protocols, such as Bluetooth, RFID, and 6LowPAN. Given the frequent use of these devices in different locations, there is a notable risk of node capture by physical access or the introduction of fake nodes [19]. These systems are vulnerable to side-channel attacks, replay attacks, malicious data injection, and routing weaknesses.

### 2.2 Network layer

The network layer is responsible for ensuring the efficient transmission and routing of data and information. The system utilizes IPv6, WiFi, 3G, GSM, and other communication protocols. Network vulnerabilities can potentially arise via data transfers and other associated procedures [20]. Unauthorized access, Denial of Service (DoS), Distributed Denial of Service (DDoS), and Man in the Middle (MitM) attacks are some of the vulnerabilities. There are also connectivity problems like lower Quality of Service (QoS) and data integrity breaches.

### 2.3 Data processing layer

The middleware, sometimes referred to as the data processing layer, is found between the network and application layers in the cloud’s three-tiered architecture. The middleware layer is positioned between the network and the application layers in order to perform computations and handle storage. It is a critical component of cloud infrastructure and can be a major source of vulnerability [21]. Cloud malware injection, SQL injection, and cloud flooding are just a few of the many attacks that can exploit the security of the middleware layer. It’s essential to address these vulnerabilities and implement strong security controls to protect critical data and processes. The middleware layer’s integrity is critical to the overall security of the cloud system.

### 2.4 Application layer

It provides an interface between the user’s application and the underlying network. For example, when you use a browser or email client, the application layer makes sure those applications can communicate over the network. It supports various protocols that enable specific types of data exchange. It ensures that data is formatted in a way the receiving application can understand. This includes encoding, compression, and encryption if necessary. DoS, SQL injection, and broken encryption are just a few of the many attacks that can exploit the security of this layer [22].

## 3 Related work

We aim to provide a more thorough literature review by incorporating additional studies that align with our research on blockchain-based ZTN and FTL in Industry 5.0 security frameworks. Initially, our related work section lacked a dedicated discussion on the unique security challenges posed by Industry 5.0 IoT environments. The role of next-generation networks in automating factories and the security risks that come with places that are always connected [22]. These studies give us important information about how we need stronger, scalable, and decentralized security systems, which makes our proposed framework even more important. The previous version did not comprehensively discuss prior blockchain-based security solutions for IoT networks. The researchers in [23], which explore how blockchain enhances security in distributed IoT architectures. These studies show that blockchain is good at protecting data transmission and building trust in decentralized settings. This is another reason why we think blockchain should be a part of the proposed framework. Our original discussion of ZTN principles and intrusion detection was limited. To address this, we have included the work of the researchers [24], which discuss implementing ZTN security models in dynamic IoT environments. These studies give us important background information on how ZTN policies stop people from getting in without permission and how deep learning-based IDS make finding threats in modern cyber-physical systems easier. Our previous related work section did not sufficiently emphasize how FTL has been applied in cybersecurity. Our research now includes studies like those in [25], which show how federated learning can help protect data privacy and make models more flexible in IoT networks. These additions strengthen our argument for adopting FTL to enable decentralized yet efficient security model updates across IoT devices. While blockchain, ZTN, and federated learning have all been studied separately in the past, there is still a big need to combine them into a single security framework for Industry 5.0 IoT environments. Our work specifically addresses this gap through the following key contributions:

- In contrast to traditional centralized models, our method uses blockchain as a decentralized ledger to safely handle and record model updates in a federated learning environment. This protects the integrity of data and builds trust across IoT devices. This builds upon the foundational work of the researchers and extends it by incorporating transfer learning to improve adaptability.
- We incorporate ZTN principles to implement stringent access control mechanisms, guaranteeing that no entity receives default trust. This fixes the problems that were pointed out in the previous work, which talked about ZTN models but didn’t look into how they could be used in federated IoT security frameworks.
- We introduce an adaptive stochastic blockchain model that continuously refines its security policies based on real-time network threats and learning updates. This model builds upon the federated learning approaches discussed by researchers by incorporating blockchain for enhanced trust management.
- The ToN-IoT and BoT-IoT datasets are used in our study to test how well our model works on a number of security measures, including accuracy, precision, recall, and F1-score. Our results are much better than baseline models and do a better job of finding intrusions than existing deep learning-based methods.

## 4 ZTN overview

ZTNs, possessing the ability to autonomously configure, monitor, heal, and optimize themselves, have become a viable option to fully automate network operations [19]. Without human interaction, their primary function is to operate and execute business procedures, maintain their own functioning, overcome obstacles, and fulfill additional obligations related to the network. ZTN can be easily installed with the use of various APIs and end-to-end (E2E) programmability [26]. The automation capabilities of ZTN are crucial to evaluating and addressing customer-specific requirements and achieving the necessary QoE. In addition, end-to-end automation allows zero-trust networks to develop and enhance by integrating and modifying the necessary features needed for practical use. The integration enables the collection and analysis of real-time network data, improving security, predictive maintenance, and overall performance. By incorporating AI/ML techniques into closed-loop operations, it is possible to automate cybersecurity procedures in the field of network and service management [17]. The ZTN architecture incorporates a data analytics service that is seamlessly integrated into all management domains. This integration allows for closed-loop operations in network security and optimization. The INSPIRE-5Gplus project, funded by the European Union (EU), introduces a comprehensive autonomous security network framework model for ZTN systems [27]. A management domain refers to a grouping of resources and services that are arranged based on different limitations, such as functions, operations, and deployment. The domain integration fabric promotes intra-domain communication, while the inter-domain integration fabric permits inter-domain communication and end-to-end management domains.

### 4.1 Security architecture

The ZTN platform benchmark structure is a fundamental component in the field of cybersecurity and networking. As the world advances towards more sophisticated and automated networks, stricter security measures become more crucial [28]. The primary goal of the ZTN safety mechanisms is to create a secure and efficient network environment, with the aim of reducing the probability of human mistakes. ZTN security frameworks must meet some essential security needs [29].

- Data Protection: The architecture of ZTN must incorporate data protection measures for information during use, movement, and rest to ensure data integrity and security. ZTN frameworks are essential for guaranteeing the accuracy and privacy of data management. Furthermore, it is crucial that the ZTN design guarantees the accessibility of facilities, data from the network, and administration services [30].
- Preservation of Privacy: In order to ensure the safety of personal information, it is essential to include security-by-default and privacy-by-design elements in the ZTN framework. These rules guarantee that the structure is initially designed to prioritize privacy by disallowing user participation and automatically implementing the most stringent privacy settings.
- Enforcement of Security Policies and Access Control: By employing the ZTN architecture, authorized service users should have the capacity to grant service access and adhere to security standards [31]. This feature allows for the automated enforcement of suitable security protocols based on the security prerequisites and the present condition of certain management services. Optimizing access control procedures improves the overall security posture.
- Detection, Prevention, and Mitigation of Intrusions:The architecture should be capable of autonomously detecting, identifying, preventing, and mitigating attacks and incidents. Having these skills is crucial for quickly recognizing and minimizing potential security risks, therefore reducing the effects of cyberattacks on the system and the services it provides [32].

The architecture ensures data confidentiality, integrity, and availability during processing, transmission, and storage. Blockchain serves as a tamper-proof ledger, securely logging and validating all model updates and transactions, thereby enabling decentralized model training, ensuring that raw data remain on local devices, thus preserving privacy. The Zero-Trust model mandates strict identity verification before allowing data access, eliminating implicit trust in any entity. The framework incorporates multi-factor authentication (MFA) and role-based access control (RBAC) for service authorization. Along with blockchain-enabled smart contracts, security policies are dynamically enforced, ensuring only authenticated and authorized entities can access Industrial IoT networks. ZTN continuously monitors network behavior and updates access control policies based on real-time security analytics. The IDS, powered by DL and FTL, continuously learns from security logs to detect anomalous patterns and emerging threats. Blockchain’s decentralized consensus mechanism ensures that model updates are verified and protected from adversarial manipulation. Adaptive security updates ensure that the framework evolves to counter zero-day attacks and advanced persistent threats (APTs).

## 5 Proposed methodology: Adaptive stochastic blockchain model for ZTN security

**Fig 1:**
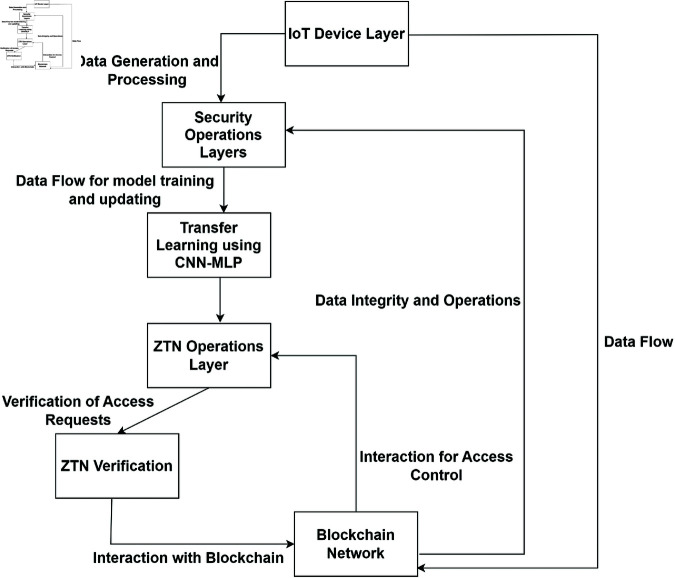
Proposed methodology adaptive stochastic blockchain model for ZTN security.

To expand on the integration of ZTN security using automated machine learning and blockchain, it will involve the development of a dynamic model that continuously updates and refines its parameters based on real-time network data and threat analysis. This approach utilizes a federated learning framework, which allows for the decentralized training of machine learning models across multiple IoT devices without the need to transfer raw data to a central location. This aligns with blockchain’s strengths in ensuring transparency, security, and data integrity. Blockchain can be used to manage and log the distribution and synchronization of the federated learning model updates. We integrate a CNN-MLP model within a deep learning-based IDS to efficiently perform feature extraction and intrusion detection. Implement smart contracts to automate the update process and ensure that only verified updates are applied to the model. This enhances security against tampering and malicious updates. Initially, each IoT device receives a copy of a pre-trained model from a central server [33]. This model is pre-trained on a large, diverse dataset that is representative of typical network behaviors and potential threats, but not specifically fine-tuned to the nuances of the individual devices’ operating environments. Each device continuously collects data relevant to its operation. This data includes both normal operational data and any anomalies or potential security threats. The types of data collected can vary widely, depending on the device’s purpose and environment, but generally include network traffic, sensor outputs, system logs, and any other telemetry data relevant to the device’s function. Before training, the device processes the collected data to extract and select the characteristics that are most indicative of its operational state and potential security risks [34]. This step is crucial because it determines what aspects of the data the model will focus on during training. Effective feature extraction and selection can significantly enhance the model’s ability to detect subtle or complex anomalies that might indicate a security threat. With the relevant features selected, each device begins adapting the pre-trained model using its local dataset. This adaptation is performed using transfer learning techniques, where the initial layers of the model (trained to recognize general patterns and features in the data) are usually kept fixed, and the deeper layers are fine-tuned to align closely with the device’s specific data characteristics. Fine-tuning involves adjusting the weights of the pre-trained model using the local data. The learning process typically focuses on the final layers of the model, where the representations are more specific to the task, accurately identifying potential security threats from normal behavior. The extent of fine-tuning can vary; in some cases, only the output layers are adjusted, while in more complex scenarios, several deeper layers might also be fine-tuned. Choice of loss function can significantly affect the model’s performance, with common choices including cross-entropy for classification tasks or mean squared error for regression tasks. Optimization is typically performed using stochastic gradient descent or one of its variants, adjusted for the limited computational resources available on many IoT devices [35]. After an initial round of training, the device evaluates the adapted model against a separate portion of its collected data that was not used during training. This step is crucial to ensure that the model not only memorizes the training data but actually learns to generalize from it [36]. Based on the performance in the validation dataset, further training iterations might be performed, tweaking the model parameters, or even revisiting the feature selection step if necessary [37, 38]. Fig 1 shows the proposed methodology. This process of local training using transfer learning enables each device in the IoT network to have a model that is both robust in its general capabilities and highly specialized to its particular needs. The result is a better overall security posture, with each device better equipped to detect and respond to the specific types of threat it is most likely to encounter in its operating environment.

Let θ represent the parameters of the global model. The initial parameters $\theta_{0}$ are derived from the pre-trained model. Each device i trains the model locally based on its dataset *D*_*i*_. The objective is to minimize a loss function *L*_*i*_ specific to the data:

$$\theta_{i}^{\text{new}}=\theta -\eta \nabla L_{i}(\theta ,D_{i})$$ (1)

In the previous equation, $\nabla L_{i}(\theta ,D_{i})$ is the gradient of the local loss with respect to the model parameters, and η is the learning rate, which controls the step size of the update.

The parameter updates from each device are aggregated to update the global model:

$$\theta^{new}=\theta +\sum_{i=1}^{n}\omega_{i}(\theta_{i}^{new}-\theta )$$ (2)

where $\omega_{i}$ represents the weight of the i-th device’s contribution, typically based on the size of *D*_*i*_ or the reliability of the device. This process repeats across several iterations t, refining the global model with each cycle:

$$\theta_{t+1}=\theta_{t}+\sum_{i=1}^{n}\omega_{i}(\theta_{i,t}^{new}-\theta_{t})$$ (3)

A mathematical model involving concepts from adaptive learning and stochastic optimization. Let *S*_*t*_ represent the state of the network at time t, which includes vectors of the network performance indicators and security metrics. Assume threats $\omega_{t}$ are stochastic processes characterized by the probability distribution that evolves over time due to external factors and adversarial strategies. Define a $\theta_{t}$ for the ML model that adapts over time based on observed data and identified threats. At each step, the model changes according to the following equation:

$$\theta_{t+1}=\theta_{t}-n_{t}\nabla_{\theta }L(S_{t},\omega_{t},\theta_{t})$$ (4)

where $\theta_{t}$ is the parameter vector at time t. *n*_*k*_ is the learning rate at time t, potentially adaptive. L is the loss function that quantifies the deviation from optimal security settings, incorporating both performance and security metrics. $\nabla_{\theta }L$ is the gradient of the loss function with respect to the model parameters.

Given the potential unavailability of explicit forms of threats and their evolution, the gradient $\nabla_{\theta }L$ is estimated using stochastic approximation methods

$$\nabla_{\theta }L=\frac{1}{N}\sum_{t=1}^{N}\nabla_{\theta }l(S_{t},\omega_{t},\theta_{t})$$ (5)

Here, l represents the loss associated with the individual samples $\left(S_{t}\;+\;1,\omega_{t}\;+\;1\right)$ drawn from the distribution of network states and threats at time t. N is the number of samples used for the estimation, which could be adapted based on the variance of the gradient estimates.

To accommodate the changing dynamics of the network and threats, the learning rate *n*_*k*_ is adjusted using an adaptive strategy based on the variance of the loss gradient:

$$n_{k}=\frac{\alpha }{{\sqrt{V}}_{t}+\epsilon }$$ (6)

α is a scaling factor, $V_{t}$ is the estimated variance of the loss gradients, and ϵ is a small constant to avoid division by zero.

Incorporate a feedback loop that adjusts the model based on discrepancies between expected and actual network performance outcomes as depicted below:

$$\theta_{t+1}=\theta_{t}-n_{k}\nabla_{\theta }L+k(S_{target}-S_{t})$$ (7)

k is a feedback gain parameter. *S*_*target*_ represents target states of network performance and security metrics. The primary objective of the proposed approach is to dynamically optimize the use of computational and communication resources in a smart factory environment. The mathematical model is formulated as follows:

$$Minimize(F(x))=\sum_{i=1}^{n}c_{i}x_{i}$$ (8)

Where F(x) is the total cost function of allocating network resources. *c*_*i*_ represents the cost associated with the resource i, which is allocated. *x*_*i*_ is a binary variable that represents whether resource i is allocated. Here are the following constraints:

- Resource Capacity
  $$\sum_{j=1}^{m}a_{ij}x_{j}\leq b_{i}$$ (9)
  where *a*_*ij*_ represents the amount of resource i required by task j. *b*_*i*_ is the total available capacity of the resource i.
- Quality of Service (QoS)
  $$QoS_{j}(x)\geq QoS_{min,j}\forall j$$ (10)
  Where: *QoS*_*j*_(*x*) is the quality of service for task j, which depends on the allocated resources. *QoS*_*min*_,*j* is the minimum quality of service required for task j.
- Latency Requirements:
  $$L(x)\leq L_{max}$$ (11)
  Where: L(x) represents the network latency under the current allocation x. *L*_*max*_ is the maximum allowable latency.

Algorithm 1 presents the algorithm for the proposed methodology.

**Algorithm 1. Adaptive stochastic blockchain model for ZTN security.**

Input: Pre-trained global model parameters global $\theta_{global}$, set of all IoT devices D, learning rate η, blockchain ledger β.

Output: Updated global model parameters global $\theta_{global}$, updated blockchain ledger β.

1: $\theta_{global}\leftarrow$DistributeGlobalModel()

β←InitializeBlockchain($\theta_{global}$)

2: For each device i∈D

$D_{i}\leftarrow$CollectLocalData(i)

$\theta_{i}^{new}$ ←LocalAdaptation $(\theta_{global}$, *D*_*i*_,η)

$\Delta \theta_{i}\leftarrow \theta_{i}^{new}-(\theta_{global})$

TransmitToBlockchain(B,i,∇ $\theta_{i}$)

3: WaitForAllUpdates(D)

B←UpdateBlockchain(B, $\theta_{global}^{new}$)

$\theta_{global}\leftarrow \theta_{global}^{new}$

4: For each access request r from any device i:

$Access_{i,r}\leftarrow$VerifyAccess(B,i,r)

LogAccessRequest(B,i,r,*Access*_*i*,*r*_ )

5: DistributeUpdatedModel(D,$\theta_{global}$)

MonitorSystem // Optional: Adjust η based on performance metrics

## 6 Results and discussion

**Fig 2:**
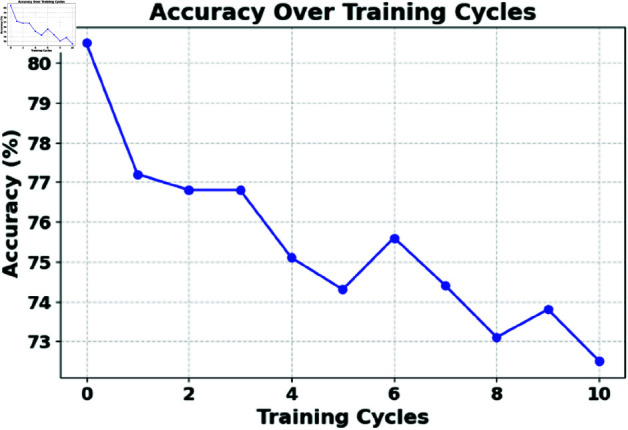
Model accuracy with training samples.

The experiment was carried out on a Dell PowerEdge T20 server with an Intel Xeon(R) CPU E3-1225v3 3.20 GHz × 4 processor. The research employs the ToN-IoT [ https://research.unsw.edu.au/projects/toniot-datasets] and BoT-IoT [https://research.unsw.edu.au/projects/bot-iot-dataset] datasets. Detailed explanations of both datasets can be found in [36–41]. The hyperparameters were found using the random search method from the scikit-learn and Keras libraries. In this study, macro-averaging methods were used to obtain the following metrics: Accuracy (AC), Detection Rate (DR), Precision (PR), F1 score. Table 1 shows the impact of learning rates and epochs on multiple model metrics. Fig 2 shows the accuracy of the model with training samples. The training curve exhibits an initial phase of rapid improvement, followed by a gradual stabilization, with slight fluctuations indicative of adjustments to the learning rate and potential overfitting. To enhance model convergence and generalization, future work will focus on adaptive learning rate scheduling, regularization techniques such as dropout and batch normalization, and hyperparameter tuning for optimal training stability. Furthermore, investigating ensemble learning strategies and reinforcement learning-based optimization could further improve the robustness of the model in dynamic IoT security environments.

**Table 1:** Impact of varying learning rates and epochs on multiple model metrics.

*Learning Rate*	*Metric*	*10 Epochs*	*50 Epochs*	*100 Epochs*
0.01	Accuracy	0.75	0.82	0.85
0.01	Precision	0.74	0.81	0.84
0.01	Recall	0.72	0.79	0.82
0.01	F1-Score	0.73	0.80	0.83
0.001	Accuracy	0.68	0.84	0.88
0.001	Precision	0.67	0.83	0.87
0.001	Recall	0.66	0.82	0.86
0.001	F1-Score	0.65	0.81	0.86
0.0001	Accuracy	0.55	0.79	0.87
0.0001	Precision	0.54	0.78	0.86
0.0001	Recall	0.53	0.77	0.85
0.0001	F1-Score	0.52	0.76	0.85

### 6.1 Impact of learning rate on model convergence

**Fig 3:**
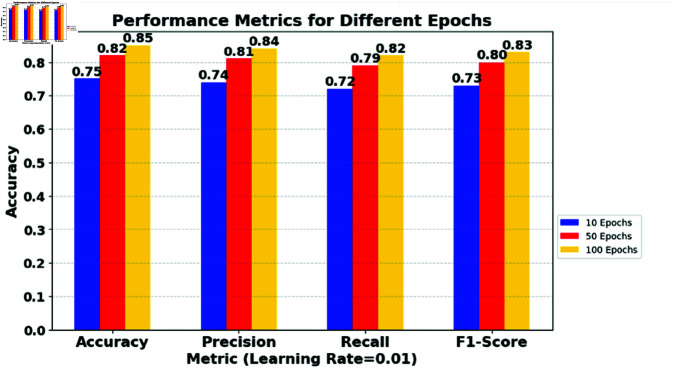
Impact of learning rate (0.01) and epochs on multiple model metrics.

**Fig 4:**
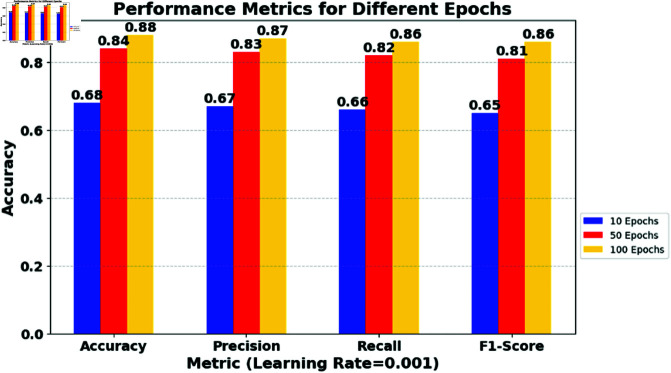
Impact of varying learning rate (0.001) and epochs on multiple model metrics.

**Fig 5:**
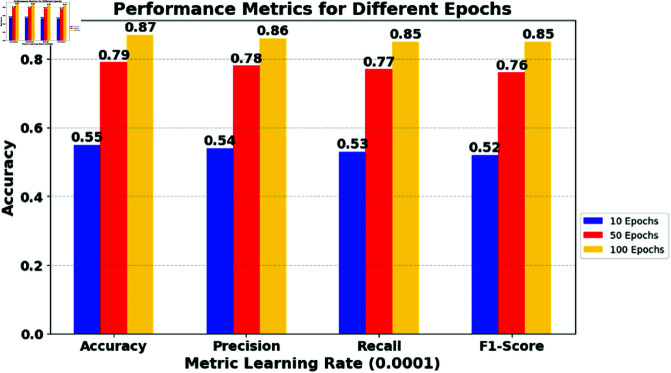
Impact of varying learning rate (0.0001) and epochs on multiple model metrics.

- High Learning Rates: The data show relatively quick improvement in all metrics as the epochs increase from 10 to 100. This suggests that a higher learning rate might lead to faster convergence in this scenario. However, it risks overshooting the loss function’s minimum, potentially causing instability if unchecked. Fig 3 shows the impact of the learning rate (0.01) and the epochs on multiple model metrics.
- Medium Learning Rates: Here, the model shows a steady improvement and eventually surpasses the performance of the higher learning rate in 100 epochs. This indicates a more stable learning path and suggests that, for longer training periods, a medium learning rate might be more effective. Fig 4 shows the impact of the learning rate (0.001) and the epochs on multiple model metrics.
- Low learning rates: The slowest improvement rate is observed with the lowest learning rate. Although initial performance is significantly lower, the final metrics at 100 epochs are competitive, indicating that given sufficient training time, lower learning rates can achieve similar, if not better, performance without risking the stability seen in higher rates. Fig 5 shows the impact of the learning rate (0.0001) and the epochs on multiple model metrics.

### 6.2 Evaluation of model metrics over epochs

- Accuracy and F1-Score: These metrics provide a comprehensive view of model performance, incorporating both the precision and recall of the model. The improvement in F1-Score is particularly critical as it balances precision and recall, providing a more holistic view of model performance.
- Precision and Recall: These metrics are especially important when the costs of false positives and false negatives are different. For instance, in a medical diagnosis scenario, a high recall might be more desirable than high precision. The data shows that precision and recall can vary differently under different learning rates, suggesting a trade-off based on the specific application requirements.

For example, a higher learning rate facilitates quicker convergence, as evidenced by the rapid improvement in all metrics from 10 to 100 epochs. This could be beneficial for scenarios where quick model deployment is necessary. However, the potential risk here is the overshooting of the minimum value of the loss function, which could lead to model instability. This is a typical trade-off seen with high learning rates, where the model may not generalize well on unseen data due to potential overfitting. However, a medium learning rate demonstrates a more gradual but consistent improvement, ultimately exceeding the performance of the higher learning rate at the 100-epoch mark. This means that the learning path is more stable, which is better for situations where model reliability is very important, like in medical or financial settings where mistakes can have significant effects. The lowest learning rate initially shows the slowest improvement, but achieves competitive performance metrics by the 100th epoch. This slow but steady approach might be ideal when the utmost accuracy is required, allowing the model to thoroughly learn the nuances of the data without the risk of rapid overfitting. From a practical point of view, these data can guide decisions regarding the appropriate learning rate and duration of training to optimize the performance of the model for specific needs. For instance, we can make adjustments in applications that prioritize precision over recall, or vice versa. The table also underscores the importance of balancing precision and recall, particularly through the F1 score, which provides a holistic measure of model performance.

## 7 Conclusion and future work

### 7.1 Conclusion

The proposed framework integrates blockchain, federated transfer learning, and zero trust network principles to enhance IoT security in Industry 5.0. By leveraging blockchain’s decentralized nature, the system ensures secure model updates and data integrity. Federated transfer learning enables privacy-preserving model training across distributed IoT devices, while the zero trust network model enforces strict access control, assuming no entity is inherently trustworthy. Experimental results using ToN-IoT and BoT-IoT datasets confirm the effectiveness of this approach, demonstrating high accuracy, precision, recall, and F1-scores across different learning rates and training epochs. The proposed framework allows addressing growing cybersecurity threats, making it a robust solution for securing IoT environments in industrial automation and smart factory applications. This research highlights the potential of combining these technologies to create a scalable and resilient security framework for next-generation industrial networks.

### 7.2 Future directions

In future work, extensive testing will be conducted on real-time data sets to evaluate the performance of the proposed framework in different IoT architectures and network conditions. In addition, we are exploring advanced techniques such as self-supervised learning, transformer-based architectures, and graph neural networks to improve anomaly detection in complex IoT environments. Explainable AI will be integrated to provide clear, transparent cybersecurity decision-making. To make the proposed framework more practical for large-scale IoT deployments, we will incorporate Hierarchical Federated Learning and lightweight blockchain features to improve efficiency. Energy-efficient deep learning models will be developed to ensure compatibility with resource-limited IoT devices. Finally, reinforcement learning will be explored to enable the real-time adaptation of security policies in response to growing cyber threats.
