# Correction: Blockchain-based zero trust networks with federated transfer learning for IoT security in industry 5.0

**DOI:** 10.1371/journal.pone.0333520

**Published:** 2025-09-29

**Authors:** Ankita Sharma, Shalli Rani, Wadii Boulila

The Funding statement for this article is incorrect. The correct Funding statement is as follows: The authors would like to acknowledge the support of Prince Sultan University for paying the Article Processing Charges (APC) of this publication.
